# Semantic Differential Scale Method Can Reveal Multi-Dimensional Aspects of Mind Perception

**DOI:** 10.3389/fpsyg.2016.01717

**Published:** 2016-11-02

**Authors:** Hideyuki Takahashi, Midori Ban, Minoru Asada

**Affiliations:** ^1^Graduate School of Engineering Science, Osaka UniversityOsaka, Japan; ^2^Faculty of Psychology, Doshisha UniversityKyoto, Japan; ^3^Graduate School of Engineering, Osaka UniversityOsaka, Japan

**Keywords:** mind perception, non-living entities, robots, semantic differential scale method, agency, experience, animism

## Abstract

As humans, we tend to perceive minds in both living and non-living entities, such as robots. From a questionnaire developed in a previous mind perception study, authors found that perceived minds could be located on two dimensions “experience” and “agency.” This questionnaire allowed the assessment of how we perceive minds of various entities from a multi-dimensional point of view. In this questionnaire, subjects had to evaluate explicit mental capacities of target characters (e.g., capacity to feel hunger). However, we sometimes perceive minds in non-living entities, even though we cannot attribute these evidently biological capacities to the entity. In this study, we performed a large-scale web survey to assess mind perception by using the semantic differential scale method. We revealed that two mind dimensions “emotion” and “intelligence,” respectively, corresponded to the two mind dimensions (experience and agency) proposed in a previous mind perception study. We did this without having to ask about specific mental capacities. We believe that the semantic differential scale is a useful method to assess the dimensions of mind perception especially for non-living entities that are hard to be attributed to biological capacities.

## Introduction

Legend has it that Saint Francis of Assisi thought that, like humans, all non-human animals, had minds and he communicated with them (Zanker, 2007). It is not only saints who perceive minds in non-human animals but the rest of us often do so on a day-to-day basis in different animals, such as monkeys, dogs, cats, and so on. Mind perception is not limited to living creatures. A wide variety of artificial entities (e.g., interactive robots) and natural phenomenon (e.g., the north wind and the sun in an Aesop’s fable) are sometimes treated as having a mind. This does not, however, mean that people of different ages and cultures share the same type of concept of the mind and the same attitudes toward the mind. For example, some Japanese people, following traditional Japanese conventions, believe that material objects (e.g., dolls and scissors) that have been used for a long time, develop minds and these people often hold a memorial ceremony for these objects when they dispose of them. By contrast, this idea that “inanimate objects have minds” is hard to make sense of in traditional Christian culture because a mind is considered a special gift from God, and artificial entities are denied a mind. Hence, it is important to investigate how we perceive minds in living and non-living entities for the purpose of understanding the diversity of human cultures (Kraft, 1995).

Questionnaire assessments are mainly used in the study of mind perception. One of the landmark studies of mind perception, conducted by Gray et al. (2007), used a large-scale web survey to analyze mind perception styles in over 2000 respondents. In this study, subjects were asked to rate the degree to which each of the 18 mental capacities (e.g., capacity to feel hunger) was suitable for explaining each of the 13 target characters (e.g., adult males, infants, dogs, and gods) on a 5-point Likert scale. The result of the study was that two orthogonal mind dimensions, named “experience” and “agency,” respectively, were found by principle component analyses (PCA). The dimension of “experience” indicates the capacity to sense and feel emotions, whereas the dimension of “agency” indicates the capacity to plan and execute intentional actions. For example, according to Gray et al.’s survey, we perceive strong “experience” but not “agency” in babies and other animals; on the contrary, we perceive “agency” but not “experience” in robots and gods. The multi-dimensional views of mind perception have been confirmed by other studies and several derivative findings, leading to the agreement that this is a good framework for explaining psychological phenomena, such as the “uncanny valley” and cognitive distortions of psychiatric disorders (Gray et al., 2011; Gray and Wegner, 2012).

Although the two mind dimensions proposed by Gray and colleagues are insightful, it is not easy to apply the questionnaire they used to measure mind perception toward non-living entities, e.g., robots. In their questionnaire, subjects were instructed to rate the degree to which a mental (biological) capacity was matched with a character. For example, subjects were asked “To what degree does a robot have a capacity to feel hunger?” The questionnaire, however, does not differentiate between what impression we have of and what we know about an entity. The subjects’ rating may be heavily influenced by their prior knowledge about robots, e.g., knowledge that robots are not capable of feeling hunger. To differentiate between what we mean by “impression” and “knowledge” of an entity, imagine the case in which a person meets a human-like android and has a strong first impression that the android feels hunger. In this case, one may perceive a mind in the android. At the same time, however, one knows that the android is a machine and does not have the capacity to feel hunger. This knowledge may prevent a person from forming a spontaneous mind perception. Most items included in Gray and colleagues’ questionnaire are concerned with mental capacities that only biological entities have. Subjects arguably have knowledge that these capacities are not implemented in non-living entities, and are forced to answer the questionnaire in a biased way. Sometimes we inevitably form an impression of an object and we assume the behavior comes from a mind. Hence, we need to exclude the effects of prior knowledge to investigate mind perception toward non-living entities, insofar as mind perception is associated with impression. The questionnaire used by Gray et al. is designed to measure one’s general conceptions of mental capacities of entities. A different questionnaire may be appropriate for assessing our mind perception.

We suggest that a semantic differential scale method could reveal multidimensional aspects of mind perception (Bradley and Lang, 1994). This scale does not include questions concerning mental capacities and is capable of assessing subject’s non-verbal impressions of various objects, events, and concepts on the basis of how they rate the matching between multiple adjectives and entities. In this method, an adjective is paired with its antonym and the two adjectives are assigned numbers on a scale. For example, “cold” is paired with “warm,” and they are given 1 and 7, respectively. Subjects were asked to evaluate where an entity is placed on the scale. In a previous study, we found that mind perception varies along two mind dimensions by using a questionnaire that included 21 paired-adjectives (Takahashi et al., 2014). The questionnaire we used was limited in generality and target; it mainly focused on brain activities in mind perception. The sample size in this previous study was small (*n* = 20) and the mean age of sample (university students) was a little biased. More importantly, we did not show that the two dimensions we found (i.e., mind-holderness and mind-readerness) corresponded to the two dimensions of “experience” and “agency” introduced by Gray and colleagues. By showing that the former dimensions correspond to the latter, we propose that our semantic differential scale is an effective way to detect the dimensions of “agency” and “experience” of mind perception.

In the current study, we performed a large-scale web survey in subjects of varying ages to generalize the two dimensions of our questionnaire. In this survey, subjects evaluated seven target characters by both the questionnaires of Gray et al. (2007) and of Takahashi et al. (2014). The results of the study suggest that the two dimensions found in our questionnaire correspond to the two mind dimensions of “experience” and “agency.” Our semantic differential scale can therefore be regarded as an effective way to intuitively assess multidimensional aspects of mind perception without asking questions about evident mental capacities.

## Materials and Methods

### Participants

Five hundred healthy Japanese subjects were recruited through an Internet survey service (Cross Marketing Co., Japan). The ages of subjects were uniformly distributed in the range of 17–75 years (mean = 45.0, *SD* = 18.9) and the gender ratio of subjects were equally divided regardless of their ages. This study was carried out with written informed consent from all subjects in accordance with the Declaration of Helsinki.

### Procedures

Subjects were asked to evaluate mind capacities and impressions of seven targets, respectively (an adult friend, a baby, a frog, a tree, a communication robot, a super computer and a god) by using both the questionnaires by Gray et al. (2007) and by Takahashi et al. (2014) on the internet website specially designed for this survey. Information of these targets was presented only using words, without pictures and the orders of the target presentation were randomized among subjects. In the questionnaire used by Gray et al. (2007), the measurement included 18 questions and subjects rated the degree to which eighteen mental capacities were suitable to explain the capacity of a target character on a 5-point Likert scale where 1 was “not suitable” and 5 was “very suitable.” In the questionnaire used in Takahashi et al. (2014), 21 pairs of two opposing adjectives were presented and subjects rated on a 7-point Likert scale ranging from 1 (a left side adjective is well matched) to 7 (a right side adjective is well matched) to express the suitable impression of target characters.

## Results

We performed a PCA for rating scores of the two questionnaires to identify dimensions of each. PCA is a statistical procedure for an orthogonal transformation to convert a set of original multidimensional data into small number of orthogonal factors called principal components (details are seen in Holand, 2008). This method is suitable to extract specific factors related to mind perception from multiple questions in questionnaires. We found two factors with eigenvalues over 1.0 in the questionnaire by Gray et al. (2007), a factor corresponding to “Experience” (eigenvalue = 11.4) accounted for 63.4% and a second factor, “Agency” (eigenvalue = 2.2), accounted for 12.3% of the variance (detail loads of questions in each component are reported in **Table 1**). Furthermore, three factors with eigenvalues over 1.0 were found in the questionnaire used by Takahashi et al. (2014) a factor named “Emotion” (eigenvalue = 11.6) accounted for 55.1% of the variance, a second factor named “intelligence” (eigenvalue = 3.0), accounted for 14.3% of the variance and a third factor (eigenvalue = 1.0), accounted for 4.9% of the variance (detail loads of questions in each component are reported in **Table 2**). Results of semantic differential scale methods are often compressed into three components by using factor analysis. However, there were no significant correlations between this third component and the other two components in Gray et al. (2007). Hence, we do not discuss the third component in this paper.

**Table 1:** Two components in Takahashi et al., 2014 (eigenvalues over 1.0).

Mental Capacity	1st (experience)	2nd (agency)
Hunger	0.679	-0.567
Fear	0.765	-0.485
Pain	0.761	-0.522
Pleasure	0.860	-0.241
Rage	0.859	-0.233
Desire	0.872	-0.166
Personality	0.842	-0.040
Consciousness	0.885	-0.073
Pride	0.836	0.146
Embarrassment	0.852	-0.127
Joy	0.866	-0.210
Self-control	0.753	0.400
Morality	0.755	0.392
Memory	0.620	0.472
Emotion recognition	0.840	0.200
Planning	0.651	0.585
Communication	0.788	0.262
Thought	0.778	0.419


**Table 2:** Three components in Takahashi et al., 2014 (eigenvalues over 1.0).

Left adjective	Right adjective	1st (emotion)	2nd (intelligence)	3rd
Mechanical	Humanlike	0.757	-0.235	-0.332
Unintelligent	Intelligent	0.632	0.489	-0.077
Unethical	Ethical	0.687	0.537	-0.040
Bad	Nice	0.871	0.142	-0.110
Uncute	Cute	0.763	-0.353	-0.213
Unfriendly	Friendly	0.816	-0.299	-0.142
Inactive	Active	0.823	-0.256	-0.066
Negative	Positive	0.858	-0.118	-0.086
Unkind	Kind	0.854	0.233	-0.097
Cold	Warm	0.831	-0.151	-0.132
Uncurious	Curious	0.842	-0.216	-0.179
Shortsight	Longsight	0.814	0.343	0.015
Emotionally unstable	Emotionally stable	0.691	0.463	0.269
Irrational	Rational	0.727	0.534	0.144
Irresponsible	Responsible	0.736	0.485	0.086
Unbiological	Biological	0.565	-0.586	0.311
Unconscious	Conscious	0.808	-0.235	0.060
Irregular	Regular	0.589	0.400	0.357
Unnatural	Natural	0.515	-0.509	0.472
Complex	Simple	0.503	-0.422	0.444
Unemotional	Emotional	0.732	-0.378	-0.128


**FIGURE 1:**
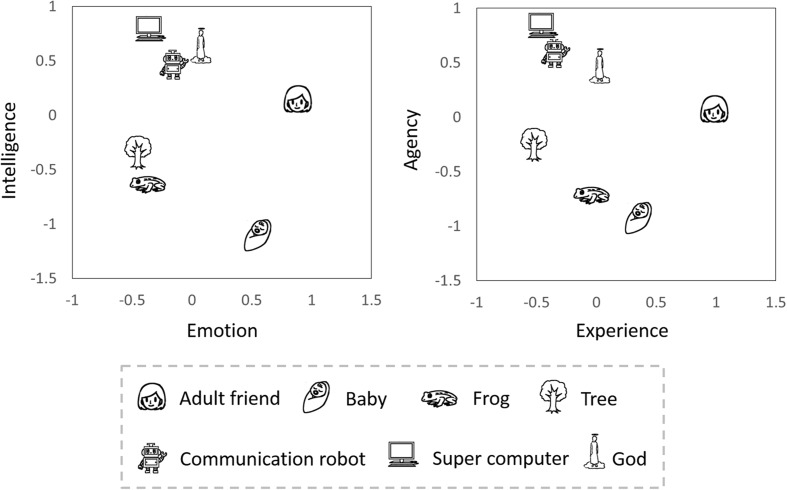
**Locations of target characters in two mind dimensions**.

We plotted mean scores of the PCA of the two questionnaires (**Figure 1**). We found that the locations of all characters in the two dimensions were similar between the two questionnaires. We calculated the correlation coefficients between “experience” and “emotion” and between “agency” and “intelligence,” respectively. Both values are positive and stochastic (“experience” and “emotion” *r* = 0.80, *p* < 0.00001, “experience” and “emotion” *r* = 0.75, *p* < 0.00001) and we concluded that the two dimensions revealed by the semantic differential scale method were similar to the two dimensions revealed from questions about mental capacities.

Furthermore, we investigated whether subject’s ages were correlated with these PCA components in each target and we found there were no significant correlations between ages and any dimensions of mind perception.

## Discussion

In this study, we directly compared two different types of questionnaires used for assessing mind perception. One asked about mental capacities and the other asked about impressions of targets by using the semantic differential scale method. From our results, we suggest that the two mind dimensions “emotion” and “intelligence” revealed in our questionnaire correspond to the dimensions “experience” and “agency” in Gray et al. (2007) questionnaire. This means that we can assesses mind perception without asking questions about mental capacities but by using the semantic differential scale method.

We consider the semantic differential scale method useful, especially when it comes to assessing multidimensional aspects of mind perception for artificial agents such as robots that are difficult to attribute mental (biological) capacities to but that we sometimes feel have a mind. Various new types of robots are being developed that are expected to communicate with us as living partners (Imai et al., 2003). We also know that attributing minds to inanimate agents improves trustworthiness and empathy toward these agents (Riek et al., 2009; Harrison and Hall, 2010; Waytz et al., 2014). These social emotions are essential to creating a good rapport between human and artificial agents. Hence, when we develop a social agent, it is important to evaluate how people perceive the mind in the agent. As our questionnaire does not include questions about mental capacities, our questionnaire can be broadly applied to the assessment of mind perception for various agents in human-robot or human-agent interaction studies.

Our study can be considered a Japanese retest of Gray et al. (2007) mind perception survey. Although the results obtained by Gray et al. (2007) and our results are relatively similar, results in artificial, inanimate objects were different between these two surveys. In our study, scores of “agency” (intelligence) in robots and super computers are higher than those in adult humans. Contrastingly, the scores of agency in robots were underestimated in Gray et al. (2007, 2011). We hypothesize that Japanese people might attribute stronger agency (intelligence) to artificial entities when compared to westerners. In some cultures, including Japan, people tend to believe non-living things have a mind, even if these things cannot be attributed to evident biological capacities. Furthermore, regardless of culture, young children tend to attribute mental states to non-living entities. For example, many children treat stuffed animals as their friends (Moriguchi et al., 2015). Moreover, some children have invisible friends called “imaginary companions” and communicate with them much like with actual human friends (Moriguchi and Shinohara, 2012). These beliefs about minds in non-living entities are often called “animism” (Harvey, 2005). Animism is the cultural attitude toward nature and external objects. Hence, this concept is strongly linked to views of life and religions in various cultures and the assessment of mind perception is quite important to understand cultural difference of these views. However, the sense of animacy is intuitive feeling and this sense cannot be explained logically. Therefore, the questionnaire that directly asks about mental capacities might not be appropriate for the assessment of mind perception in animism culture. We believe that the assessment of mind perception by using the semantic differential scale method is an intuitive way to assess subject’s impressions and this method is suitable to assess mind perception universally regardless of cultural differences. Further, because the abilities for processing other’s mind are often distorted in various psychiatric disorders (e.g., schizophrenia), our intuitive method might be useful for assessing these patient’s symptoms (Matsumoto et al., 2015).

## Author Contributions

HT, MB, and MA designed the research. HT and MB performed the research. HT wrote this paper.

## Conflict of Interest Statement

The authors declare that the research was conducted in the absence of any commercial or financial relationships that could be construed as a potential conflict of interest.
